# Supplementary material for: Synergistic Effects of Scalp Acupuncture and Repetitive Transcranial Magnetic Stimulation on Cerebral Infarction: A Randomized Controlled Pilot Trial
Source: Brain Sci. 2020 Feb 7;10(2):87. doi: 10.3390/brainsci10020087 (PMC7071610; doi:10.3390/brainsci10020087)
Supplement: Supplementary file 1 [file brainsci-10-00087-s001.pdf]

Supplementary Table S1. CONSORT 2010 checklist of information to include when reporting a randomized trial.

CONSORT 2010 checklist of information to include when reporting a randomised trial*

Section/Topic	Item No	Checklist item	Reported on page No
**Title and abstract**			
	1a	Identification as a randomised trial in the title	1
	1b	Structured summary of trial design, methods, results, and conclusions (for specific guidance see CONSORT for abstracts)	1
**Introduction**			
Background and objectives	2a	Scientific background and explanation of rationale	1-2
	2b	Specific objectives or hypotheses	2
**Methods**			
Trial design	3a	Description of trial design (such as parallel, factorial) including allocation ratio	2
	3b	Important changes to methods after trial commencement (such as eligibility criteria), with reasons	none
Participants	4a	Eligibility criteria for participants	4
	4b	Settings and locations where the data were collected	3-4
Interventions	5	The interventions for each group with sufficient details to allow replication, including how and when they were actually administered	4-5
Outcomes	6a	Completely defined pre-specified primary and secondary outcome measures, including how and when they were assessed	5-6
	6b	Any changes to trial outcomes after the trial commenced, with reasons	none
Sample size	7a	How sample size was determined	6
	7b	When applicable, explanation of any interim analyses and stopping guidelines	none
Randomisation:			
Sequence generation	8a	Method used to generate the random allocation sequence	4
	8b	Type of randomisation; details of any restriction (such as blocking and block size)	4
Allocation concealment mechanism	9	Mechanism used to implement the random allocation sequence (such as sequentially numbered containers), describing any steps taken to conceal the sequence until interventions were assigned	4
Implementation	10	Who generated the random allocation sequence, who enrolled participants, and who assigned participants to interventions	4
Blinding	11a	If done, who was blinded after assignment to interventions (for example, participants, care providers, those assessing outcomes) and how	4
	11b	If relevant, description of the similarity of interventions	none
Statistical methods	12a	Statistical methods used to compare groups for primary and secondary outcomes	6
	12b	Methods for additional analyses, such as subgroup analyses and adjusted analyses	none
**Results**			
Participant flow (a diagram is strongly recommended)	13a	For each group, the numbers of participants who were randomly assigned, received intended treatment, and were analysed for the primary outcome	6-7
	13b	For each group, losses and exclusions after randomisation, together with reasons	6-7
Recruitment	14a	Dates defining the periods of recruitment and follow-up	6
	14b	Why the trial ended or was stopped	6-7
Baseline data	15	A table showing baseline demographic and clinical characteristics for each group	Table 2
Numbers analysed	16	For each group, number of participants (denominator) included in each analysis and whether the analysis was by original assigned	Fig1

		groups	
Outcomes and estimation	17a	For each primary and secondary outcome, results for each group, and the estimated effect size and its precision (such as 95% confidence interval)	9-15
	17b	For binary outcomes, presentation of both absolute and relative effect sizes is recommended	none
Ancillary analyses	18	Results of any other analyses performed, including subgroup analyses and adjusted analyses, distinguishing pre-specified from exploratory	none
Harms	19	All important harms or unintended effects in each group (for specific guidance see CONSORT for harms)	15
**Discussion**			
Limitations	20	Trial limitations, addressing sources of potential bias, imprecision, and, if relevant, multiplicity of analyses	16-17
Generalisability	21	Generalisability (external validity, applicability) of the trial findings	15
Interpretation	22	Interpretation consistent with results, balancing benefits and harms, and considering other relevant evidence	15-16
**Other information**			
Registration	23	Registration number and name of trial registry	3
Protocol	24	Where the full trial protocol can be accessed, if available	Reference 25
Funding	25	Sources of funding and other support (such as supply of drugs), role of funders	17

*We strongly recommend reading this statement in conjunction with the CONSORT 2010 Explanation and Elaboration for important clarifications on all the items. If relevant, we also recommend reading CONSORT extensions for cluster randomised trials, non-inferiority and equivalence trials, non-pharmacological treatments, herbal interventions, and pragmatic trials. Additional extensions are forthcoming: for those and for up to date references relevant to this checklist, see www.consort-statement.org.

Supplementary Table S2. Revised Standards for Reporting Intervention in Clinical Trials of Acupuncture (STRICTA).

	Item Criteria	Description
1.Acupuncture rationale	1a) Style of acupuncture	Korean Medicine Therapy
	1b) Reasoning for treatment provided – based on historical context, literature sources, and/or consensus methods, with references where appropriate	1) Discussion among four doctors that practice Korean medicine (consensus)<br>2) Textbook of acupuncture and moxibustion medicine<br>3) Relevant articles<br>Selection of treatment regions based on textbooks, related papers, and expert discussions
	1c) Extent to which treatment varied	Standardized treatment
2. Details of needling	2a) Number of needle insertions per subject per session (mean and range where relevant)	2–4
	2b) Names (or location if no standard name) of points used (uni-/bilateral)	SIAN's MS6; MS7 of the lesional hemisphere
	2c) Depth of insertion, based on a specified unit of measurement or on a particular tissue level	Needles were horizontally inserted into the subcutaneous tissue of the scalp, about 3 cm deep.
	2d) Responses sought	No de qi or muscle twitching – only sensation due to needle insertion
	2e) Needle stimulation	None
	2f) Needle retention time	20-min per session
	2g) Needle type	KOS92 nonmagnetic steel disposable needles (0.25-mm diameter and 30-mm long), manufactured by Dong Bang Acupuncture, Inc.
3. Treatment regimen	3a) Number of treatment sessions	15
	3b) Frequency and duration of treatment sessions	Five times/week for 3 weeks, 20-min per session
4. Other treatment	4a) Details of other interventions administered to the acupuncture group	Conventional stroke rehabilitation therapy

components	4b) Setting and context of treatment – including instructions to practitioners – as well as information and explanations given to patients	Practitioner-patient conversation about the context of the treatment, life habits, and daily life management
5. Practitioner background	5a) Description of participating acupuncturists	Korean medicine doctor with the following qualifications: 6 years of formal university training in Korean medicine, a license, and at least 2 years of clinical experience
6. Control or comparator interventions	6a) Rationale for the control or comparator in the context of the research question, with sources that justify the choice	Wang Y, Shen J, Wang XM, Fu DL, Chen CY, Lu LY et al. Scalp Acupuncture for Acute Ischemic Stroke: A Meta-Analysis of Randomized Controlled Trials. Evid Based Complement Altern Med 2012;2012:480950.: Lee SJ, Shin BC, Lee MS, Han CH, Kim JI. Scalp acupuncture for stroke recovery: A systematic review and meta-analysis of randomized controlled trials. European J Integr Med. 2013;5:87-99
	6b) Precise description of the control or comparator; details for items 1−3 above with the use of sham acupuncture or any other type of acupuncture-like control	Conventional stroke rehabilitation therapy for control, rTMS, and SAEM-CS groups. LF-rTMS applied to the hot spot of the M1 region (the motor cortex at the contralesional hemisphere) for the rTMS group and LF-rTMS applied to the same M1 and simultaneous SA stimulation over the upper MS6 and MS7 region of the lesional hemisphere for the SAEM-CS group.

Supplementary Table S3. Changes in outcome measures after treatment completion in four group.

Groups	Dependent Variables	Week 0 (M±SD)	Week 3 (M±SD)	Week 7 (M±SD)	Defference (W3-W0)	Defference (W7-W0)	$x^2(P)$
Control group	FMA Upper Affected side	44.42±26.57	49.58±24.33	50.25±23.64	5.17±10.53	5.83±11.30	9.15 (.010)
	FMA Lower Affected side	19.33±11.22	24.50±10.41	24.67±10.19	5.17±4.17	5.33±6.40	8.83 (.012)
	FMA Total Affected side	63.75±36.00	74.08±33.22	74.92±31.13	10.33±12.87	11.17±13.07	11.87(.003)
	NIHSS	3.50±4.46	3.33±4.03	2.00±2.70	-0.17±3.46	-1.50±2.32	4.00 (.135)
	MBI	65.58±18.92	72.42±23.19	81.50±18.53	6.83±13.16	15.92±11.94	17.55 (<.001)
	FIM	93.83±16.72	101.00±20.53	107.33±17.52	7.17±13.54	13.50±11.44	6.53 (.038)
	Nine-hole	93.65±39.18	66.59±35.87	58.72±38.28	-27.06±33.08	-34.93±37.61	12.67(.002)
	AHSA-NOMS	6.83±0.58	7.00±0.00	-	0.17±0.58	-	-
	FAC	2.08±1.83	2.50±2.07	-	0.42±1.00	-	-
	mRS	3.08±0.90	3.08±1.44	2.50±1.45	0.00±1.21	-0.58±1.00	5.17 (.076)
	EQ-5D	9.75±2.73	9.25±2.14	9.08±2.97	9.83±1.64	9.67±2.61	2.33 (.311)
	K-MMSE	26.42±3.75	26.42±3.48	-	0.00±3.07	-	-
	MAS Elbow	0.08±0.29	0.17±0.39	0.17±0.39	0.08±0.51	0.08±0.51	0.40 (.819)
	MAS Ankle	0.25±0.62	0.33±0.65	0.25±0.62	0.08±0.51	0.00±0.43	0.50 (.779)
	Griptest Dominant - hand	31.17±18.15	28.92±14.32	29.50±13.28	-2.25±7.53	-1.67±7.11	0.30 (.862)

	Griptest Non Dominant – hand	13.50±15.56	15.00±12.66	18.42±13.39	1.50±9.79	4.92±16.23	0.20 (.905)
	APB recording Corticalstim latency	11.83±12.43	9.91±12.31	9.89±12.34	-1.93±6.54	-1.94±7.16	2.00 (.368)
	APB recording Corticalstim Amplitude	258.33±412.71	409.67±675.5	273.33±435.64	151.33±421.80	15.00±252.13	1.00 (.607)
	AH recording Cortical stim latency	22.68±22.58	25.94±23.05	22.03±23.08	3.27±22.33	-0.65±2.31	0.84 (.657)
	AH recording Cortical stim Amplitude	294.50±471.46	353.08±586.75	255.42±338.39	353.73±586.08	-39.08±325.71	2.77 (.250)
SA group	FMA Upper Affected side	26.18±29.20	35.27±24.41	39.36±25.24	9.09±8.60	13.18±15.72	14.00(.001)
	FMA Lower Affected side	19.27±8.97	25.27±10.11	24.55±10.53	6.00±4.67	5.27±4.41	9.14(.010)
	FMA Total Affected Side	45.45±36.05	60.55±32.15	63.91±35.02	15.09±11.89	18.45±17.01	12.05(.002)
	NIHSS	5.73±3.77	3.09±3.91	3.36±4.08	-2.64±2.69	-2.36±2.46	9.56(..008)
	MBI	55.36±23.08	69.73±29.07	74.00±29.36	14.36±11.74	18.64±18.21	11.46(..003)
	FIM	87.36±21.46	98.00±23.23	102.45±23.70	10.64±8.63	15.09±15.12	10.369(..006)
	Nine-hole	89.43±42.73	87.59±45.37	88.94±43.88	-1.84±3.63	-0.49±3.76	3.50(..174)
	AHSA-NOMS	6.45±0.93	6.73±0.65	-	0.27±1.01	-	-
	FAC	1.18±1.78	2.64±2.06	-	1.45±1.51	-	-
	mRS	3.45±0.69	3.18±1.60	2.80±1.48	-0.27±1.42	-0.91±1.76	0.50(.779)

	EQ-5D	9.73±3.41	10.00±2.14	10.55±2.46	10.91±2.34	11.45±2.42	1.56(..458)
	K-MMSE	25.00±4.56	24.91±5.75	-	-0.09±3.70	-	-
	MAS Elbow	0.45±0.93	0.55±0.69	0.36±0.50	0.09±0.54	-0.09±0.70	1.40(.497)
	MAS Ankle	0.36±0.67	0.27±0.47	0.36±0.50	-0.09±0.83	0.00±0.77	0.40(.819)
	Griptest Dominant - hand	27.91±9.68	32.55±18.45	29.36±13.17	4.64±14.29	1.45±6.58	0.61(.739)
	Griptest Non Dominant – hand	10.91±16.18	11.36±17.18	11.45±17.01	0.45±4.08	0.55±4.25	1.39(.499)
	APB recording Corticalstim latency	8.31±11.56	10.48±12.08	10.37±11.95	2.17±7.54	2.06±6.91	0.00(1.00)
	APB recording Corticalstim Amplitude	372.73±567.00	407.64±615.19	492.82±843.53	34.91±130.93	120.09±630.49	0.40(.819)
	AH recording Cortical stim latency	23.47±22.63	23.45±22.76	23.87±23.07	-0.03±21.05	0.40±20.92	0.92(.630)
	AH recording Cortical stim Amplitude	144.09±181.25	150.18±216.32	255.45±376.84	149.78±214.39	111.36±266.37	0.67(.717)
rTMS group	FMA Upper Affected side	33.13±19.40	50.13±10.78	56.50±9.70	17.00±13.89	23.38±14.70	13.61(.001)
	FMA Lower Affected side	19.63±8.77	24.50±5.86	28.25±6.78	4.88±6.49	8.63±5.24	10.13(.006)
	FMA Total Affected Side	52.75±25.02	74.63±15.22	84.88±14.24	21.88±17.67	32.13±17.27	12.25(.002)
	NIHSS	5.13±3.23	2.88±2.36	2.00±1.85	-2.25±1.75	-3.13±1.73	12.29(.002)
	MBI	41.63±22.60	67.38±19.94	85.13±11.68	25.75±10.01	43.50±16.64	15.55(<.001)

	FIM	75.38±13.20	97.75±14.74	111.50±8.49	22.38±4.66	36.13±6.88	15.55(<.001)
	Nine-hole	108.75±31.83	83.30±40.88	68.56±37.29	-25.45±34.42	-40.19±35.61	11.27(.004)
	AHSA-NOMS	5.38±1.92	6.50±0.76	-	0.13±2.03	-	-
	FAC	1.38±1.51	2.63±1.60	-	1.25±1.04	-	-
	mRS	3.63±0.52	2.88±1.36	2.63±1.06	-0.75±1.16	-1.00±0.76	7.52(.023)
	EQ-5D	12.13±1.64	10.00±2.45	9.13±1.25	11.00±2.27	10.13±1.46	11.47(.003)
	K-MMSE	26.13±3.52	26.50±3.7	-	0.38±2.07	-	-
	MAS Elbow	0.13±0.35	0.63±0.52	0.75±0.71	0.50±0.53	0.63±0.74	5.33(.069)
	MAS Ankle	0.00±0.00	0.50±0.54	0.38±0.52	0.50±0.53	0.38±0.52	5.20(.074)
	Griptest Dominant - hand	25.63±13.74	28.38±11.62	27.38±9.77	2.75±8.19	1.75±7.19	0.45(.798)
	Griptest Non Dominant – hand	6.50±9.35	9.88±7.90	7.88±5.41	3.38±6.57	1.38±7.50	0.69(.707)
	APB recording Corticalstim latency	9.45±13.12	14.79±12.35	18.29±11.44	5.34±10.58	8.84±12.66	4.26(.119)
	APB recording Corticalstim Amplitude	188.00±449.58	297.75±459.71	441.75±416.07	109.75±138.34	253.75±573.24	6.35(.042)
	AHrecording Cortical stim latency	20.53±24.30	15.91±22.13	28.36±24.11	-4.61±20.52	7.84±20.38	0.95(.623)
	AHrecording Cortical stim Amplitude	201.63±353.65	90.88±143.49	98.50±102.30	83.04±150.54	-103.13±305.58	0.11(.949)
SAEM-CS group	FMA Upper Affected side	42.64±27.21	44.91±27.72	47.64±25.32	2.27±7.28	5.00±6.80	7.60(.022)

FMA Lower Affected side	19.45±12.89	23.64±11.24	25.36±10.57	4.18±5.65	5.91±6.50	10.07(.007)
FMA Total Affected Side	62.09±39.03	68.55±38.39	73.00±35.56	6.45±12.11	10.91±12.43	9.63(.008)
NIHSS	4.18±4.77	4.00±4.02	3.64±4.48	-0.18±1.89	-0.55±1.92	2.23(.328)
MBI	57.55±30.27	73.09±25.99	80.55±27.19	15.55±14.98	23.00±16.12	17.05(<.001)
FIM	85.73±35.99	101.64±22.55	107.91±23.36	15.91±21.14	22.18±20.84	16.60(<.001)
Nine-hole	81.90±42.01	63.19±39.08	64.56±45.76	-18.70±36.07	-17.34±51.43	7.00(.030)
AHSA-NOMS	6.45±0.82	6.64±0.92	-	0.18±0.75	-	-
FAC	1.73±1.68	2.73±2.01	-	1.00±1.61	-	-
mRS	3.27±0.79	3.09±1.30	2.55±1.63	-0.18±1.08	-0.73±1.49	2.60(.273)
EQ-5D	10.09±3.27	9.00±3.13	9.45±3.70	9.73±2.65	10.18±3.19	7.40(.025)
K-MMSE	23.00±4.29	25.09±3.33	-	2.09±1.92	-	-
MAS Elbow	0.27±0.47	0.45±0.82	0.18±0.40	0.18±0.75	-0.09±0.30	1.50(.472)
MAS Ankle	0.36±0.50	0.55±0.82	0.27±0.65	0.18±0.60	-0.09±0.54	2.80(.247)
Griptest Dominant - hand	20.55±9.72	21.73±7.77	21.45±9.40	1.18±4.58	0.91±5.94	0.06(.973)
Griptest Non Dominant – hand	10.64±10.27	11.45±10.83	12.45±11.20	0.82±3.71	1.82±5.86	1.40(.497)
APB recording Corticalstim latency	12.33±11.86	14.70±11.73	16.83±10.89	2.37±12.60	4.50±10.16	1.75(.417)

	APB recording Corticalstim Amplitude	576.73±781.20	266.00±241.09	882.45±949.47	-310.73±808.53	305.73±442.54	5.25(.072)
	AHrecording Cortical stim latency	14.52±20.20	15.05±20.93	15.23±21.21	0.54±18.52	0.71±1.25	4.11(.128)
	AHrecording Cortical stim Amplitude	238.55±362.56	156.91±265.20	264.91±413.43	156.20±264.08	26.36±279.46	0.11(.949)
